# Implementation of the National Action Plan Health Literacy in Germany—Lessons Learned

**DOI:** 10.3390/ijerph17124403

**Published:** 2020-06-19

**Authors:** Doris Schaeffer, Svea Gille, Klaus Hurrelmann

**Affiliations:** 1 School of Public Health, Interdisciplinary Centre for Health Literacy Research, Bielefeld University, 33615 Bielefeld, Germany; doris.schaeffer@uni-bielefeld.de; 2 Department Public Health and Education, Hertie School, 10117 Berlin, Germany; hurrelmann@hertie-school.org

**Keywords:** health literacy, national action plan, diffusion, dissemination, implementation, collaborative approach, Germany

## Abstract

The promotion of health literacy (HL) has become an important task in public health. In response, in many countries, strategies and action plans to strengthen HL have been developed. Systematic discussion of implementation strategies of action plans on HL is scarce. This paper presents the implementation strategy and the methodical process of its realization of the National Action Plan HL in Germany which was published in 2018. The implementation strategy was based on considerations of implementation science and research. A process consisting of a continuum of various overlapping methodical and strategic steps of diffusion, dissemination and implementation based on collaboration and co-production was chosen. According to this, the Action Plan was widely diffused via various channels, disseminated through numerous publications and presentations, and implemented in several settings by holding workshops with stakeholders from politics, science and practice, as well as by cooperating with the Alliance for Health Literacy. This three-part collaborative and co-productive implementation strategy has helped to place HL and the National Action Plan on the health policy agenda in Germany. Experience demonstrates that implementation should be also considered, systematically planned, and addressed when developing strategies to strengthen HL.

## 1. Introduction

Health literacy has become an important issue in many countries, as reflected by the broad coverage in international literature and numerous WHO documents. Health literacy is understood as the “competences to access, understand, appraise, and apply health information” [1] (p. 3), important to make sound health decisions [2] in order to promote health, to take an active part in managing health and illness in everyday life, and to use the health care system. In modern digital knowledge societies, health literacy is also indispensable to orientate oneself among the abundance of mostly digital health information, to find reliable information, and to assess the quality of information, including false and incorrect information. However, available studies show that health literacy is insufficient in many countries, including Germany [3]. Poor health literacy has numerous negative consequences, ranging from negative health behavior, a higher risk of disease, less self-care, and deficits in coping with illness and chronicity to over- and misuse (extensive use) of health care [4,5]. The promotion of health literacy is therefore an important task of public health [6,7].

In many countries, strategies to improve health literacy and “National Action Plans” have been developed [8,9,10], e.g., in the United States [11], in Scotland [12], or Portugal [13], and also discussions on their implementation and efficacy started slowly [9,10]. An Action Plan was also developed in Germany, based on the example of nine countries at the time. The multi-step methodical process of developing and agreeing on the plan has already been reflected on in another publication [14]. This paper aims to present the implementation strategy and describes the methodical process of its realization. It concludes with a discussion of the experiences gained with this strategy.

## 2. Materials and Methods

### 2.1. Origin and Structure of the National Action Plan Health Literacy in Germany

The National Action Plan Health Literacy in Germany was developed immediately following publication of the first studies on the health literacy of the population [15,16,17], which showed significantly worse results than expected and caused great concern. In Germany, low levels of health literacy affect more than half the population. The study results also point to vulnerable groups with higher amounts of low health literacy. These include people with migration background, low socioeconomic status, low education, as well as older people and chronically ill people. In reaction to this, a group of proven experts from various scientific disciplines including medicine, health sciences, education, sociology, nursing science as well as from politics and selected fields of health and social services formed a civil society initiative with the objective of developing a systematic program, the National Action Plan, to strengthen health literacy in Germany. A scoping review of the literature and a content analysis of the empirical findings on health literacy, as well as existing action plans for the promotion of health literacy, revealed a large number of health literacy definitions and conceptual models. After a detailed discussion, the expert group agreed on the definition and model developed within the framework of the European Health Literacy Survey [1]. Moreover, a relational approach to health literacy, meaning the interaction between personal competencies and the demands of systems [7], was considered as important. Based on this, the expert group developed a total of 15 coordinated recommendations [18]. The recommendations show how health literacy of the German population with special attention to vulnerable groups can be improved, and which measures should be initiated to achieve this in four *areas of action*:

The *first area of action* focuses on *everyday life*. The German nationwide study on health literacy (HLS-GER) [15] shows that the population finds it particularly difficult to deal with information on health promotion and the healthy design of everyday living environments. This is true, for example, in the areas of education and training, the media, the work environment, and the leisure and consumption sector. The recommendations aim to promote health literacy in every single sector.

The *second area of action* is devoted to the *health care system*. Due to its complexity, the multitude of entities, and lack of transparency, the German health care system places very high demands on users. The recommendations aim at further developing the health care system into a user-friendly, patient-centered, and health literate health care system.

The *third area of action* focuses on *chronic health disorders and diseases*. They account for the majority of health problems and are associated with a wide range of challenges for the patients and their relatives. Such health disorders and diseases have numerous physical, social, psychological, and economic consequences. The recommendations aim to enable health literate illness- and self-management and to anchor health literacy at all levels of the health care system.

*Research* on health literacy is the focus of the *fourth area of action*. Research should be expanded in this field, and appropriate funding should be provided.

The National Action Plan was developed in just over one and a half years and submitted in 2018 to the Minister of Health, who was also the patron of the project. The National Action Plan is primarily addressed to policymakers but also to relevant actors in the health care system and other areas of society, such as the education and training system, the leisure sector, or the work sector. The overall objective of the Action Plan was to initiate an alliance that includes all sectors of society to enable a comprehensive approach to the sustainable improvement of health literacy.

### 2.2. Framing the Implementation of the National Action Plan Health Literacy in Germany

With the handover of the National Action Plan in 2018 the implementation phase began. It was largely uncharted territory. Up to now, only very few countries have had any experience with implementing such action plans within the complex structures of policy and practice. Systematic discussion of implementation strategies and aspects of action plans on health literacy is also only just beginning [9,10]. In many cases, the plans were also developed in countries where the health care system is strongly influenced by the state. Here, recommendations can be realized by superordinate bodies who formulate guidelines and also enforce and monitor their implementation [14]. However, there is less evidence about the efficiency of such top-down strategies for implementing action plans or expert recommendations. Obviously, even in centralized systems, top-down strategies are difficult and only partially effective [14,19].

In Germany, the health care system is not state-controlled, but mainly organized according to the principle of subsidiarity. This means that the state can only formulate political principles that can be realized and implemented independently by organizations and associations [14]. In order to implement the National Action Plan, it is therefore necessary to involve not only political decision-makers but also the relevant bodies, organizations, associations and actors of self-administration within the health care system in the implementation and realization of the recommendations. A bottom-up, collaborative implementation strategy is therefore appropriate.

This applies all the more so, since the National Action Plan Health Literacy in Germany was not drawn up by a group of experts appointed by government bodies but by a civil society initiative of experts from academia, politics, and health care and social organizations. Implementation therefore aimed at both politicians and important social actors from the health care system and other areas of society, such as the education and training system. Both should be motivated and encouraged to implement the Action Plan and were therefore the central target groups to which the implementation efforts were directed.

These particularities require special attention from an implementation point of view. In fact, implementation research in political science usually focuses on the execution of policy programs. The political executive branch is the central actor in this process. This is different in the case of the National Action Plan Health Literacy in Germany, because—as previously mentioned—it is not a political but a civil society initiative. This is the reason why policymakers are the target of the implementation efforts instead of the initiators of strategies or measures, as is usually the case.

### 2.3. Theoretical Considerations of the Implementation Strategy

The design of the National Action Plan’s implementation strategy was based on considerations of implementation science and research, which have been greatly enhanced in recent years. Particularly in the health sciences, implementation research based on translation, knowledge transfer and integration, and the use of empirical evidence has gained great importance. In this stream “implementation science is defined as the scientific study of methods to promote the systematic uptake of research findings and other EBPs (evidence based practice) into routine practice to improve the quality and effectiveness of health services and care” [20] (p. 2). It thus aims primarily at bridging the knowledge–practice deficit (know do gap [19]). Furthermore, implementation is seen here as a continuum consisting of diffusion–dissemination–implementation [20]. While diffusion represents the passive, non-targeted spreading of new ideas and concepts, dissemination is the active distribution to specific target groups. According to this understanding, implementation aims at the introduction of new concepts into certain settings and presupposes that there is a willingness to adopt. This *continuum* will be used for the strategy developed here.

This is complemented by implementation research in political and social sciences, which deals with the execution of political programs or laws, as well as the introduction of innovations [21]. Implementation is seen here as the process of putting a policy or political strategy into execution. The focus in this article is on this tradition, because action plans are by nature less about new evidence-based interventions to be introduced into everyday professional practice and more about new political programs that should find their way into practical politics and lead to innovation.

Different approaches can be identified in policy implementation research: Starting in the 1970′s, one important approach dealt with the gap between policy design, the outcome of policies (policy execution), and the analysis of existing implementation deficits [19,21]. Later, attention increasingly turned to the causes of this gap. It was not only the question of why policy implementations failed but also what caused implementation to fail; how existing obstacles can be overcome; and, in general, what conditions contribute to success. Top-down versus bottom-up approaches were discussed and contrasted [22]. The former was considered the reason for many of the implementation deficits. The search for ways to overcome or avoid these gaps and deficits was also of interest. An important approach to this is seen in the science of delivering results [23], to improve policy designs through deliberation (consulting).

A related approach by Ansell et al. [21] is the theory that the real obstacle to policy implementation lies in “badly designed policies”. The authors propose a policy-making model based on *collaboration* with upstream and downstream actors to prevent implementation failure [21] (p. 478). This approach is generally transferable to implementation and appears to be particularly interesting for the project in question. Another relevant aspect in this context is policy design as an ongoing process [24]. According to this, implementation is always about the introduction of innovations or changes. These changes are usually met with resistance at first [25]. They are rarely adopted ad hoc but usually step by step and should therefore be conceived as a *process*.

**Figure 1:**
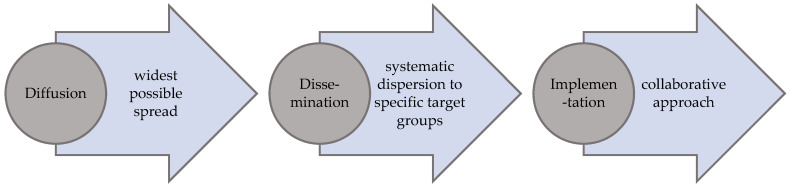
Process of implementing the National Action Plan Health Literacy in Germany.

Many of the approaches outlined here are more analytically oriented and do not aim at the question of “how to”, i.e., how implementation should take place [20] (exception [19]). However, this question actually is at the forefront of the implementation of the Action Plan (and this paper). Therefore, in order to frame the implementation strategy for the Action Plan, it’s realization is not understood as a one-off event but as a *process* consisting of a continuum of various overlapping methodical and strategic steps of diffusion, dissemination and implementation, which build upon each other [20,21] (see Figure 1).

## 3. Results

### 3.1. First Step in the Process of Realization: Diffusion

The first step aimed at the widest possible *diffusion* of the Action Plan and its recommendations. This refers to an unspecified spread of the National Action Plan. To achieve this, various channels were used to make the Action Plan known. The starting point was a major event with the participation of actors from politics, self-administration, media, and science. The Action Plan was then dispatched in great numbers by regular mail and circulated electronically via an extensive e-mail distribution list, which was later used as a medium for information and dissemination. In particular, the website has been an important medium for distributing the National Action Plan. The Action Plan can be accessed in German and English [26]. 

In addition, the website also provides background information on the Action Plan’s fields of activity and recommendations, the actors involved in its development (management team, expert advisory board, and management office), as well as on the topic of health literacy in general and on planned/implemented events. The Action Plan website has recorded almost 71,600 hits with 30,100 visitors since its relaunch in May 2018 and is listed fourth in Germany (as of 25 May 2020) for the Google search term “Gesundheitskompetenz” (the German translation of English “health literacy”) after Wikipedia, the Federal Centre for Health Education, and the Robert Koch Institute. Thus, it was distributed not only among different expert networks but also by word-of-mouth among many associations and organizations. At the same time, numerous press releases and media reports about the Action Plan were made. The 11-member expert advisory board also contributed intensively to the diffusion of the Action Plan through their own networks.

### 3.2. Second Step in the Process of Realization: Dissemination

The second parallel step focused on *dissemination*, which meant a systematic dispersion of the Action Plan to specific target groups important for implementation, mainly politicians and key stakeholders. These include political leaders at the federal and state level, and executives of associations and top organizations in the health care sector, as well as of welfare organizations or the education and training sector, donors, and foundations. Empirical studies on their relevance for implementation are available [19]. The aim was to disseminate the Action Plan to these target groups, to inform them about the relatively new topic of health literacy and the recommendations of the Action Plan, and to convince them to support the promotion of health literacy.

Dissemination took place through publications in relevant journals and presentations at conferences and congresses hosted by organizations and professions of the health care system and other relevant areas of society. In the two years following publication of the Plan, a total of almost 55 presentations were given at national and international congresses and a wide variety of specialist events. Further presentations were given by members of the expert group in their respective contexts and networks. In addition, 20 journal articles were prepared and published, including a series of articles on the Action Plan and its recommendations in a German health services research journal [26].

### 3.3. Third Step in the Process of Realization: Collaborative Implementation

The third step was dedicated to *implementation*: the introduction of the Action Plan in several important arenas with a collaborative approach. For this purpose, an approach based on collaboration and co-production was chosen, focusing on (a) workshops with stakeholders and important actors from politics, science, and practice and (b) participation in the Alliance for Health Literacy.

#### 3.3.1. Workshops

The workshops form the core element of the collaborative implementation strategy for the Action Plan. The aim of the workshops was to initiate a co-productive discussion and further development of the recommendations contained in the National Action Plan into concrete measures to be transferred directly to the respective setting for implementation. This was meant to promote identification with the Action Plan, as well as motivate and convince the actors involved to support its implementation within their own sphere of activity. Based on the results of the workshops, strategy papers on the specific recommendations were developed and then disseminated (electronically). Where possible, the strategy papers were then published in relevant journals in compact form.

Approximately 30–40 participants each attended the one-day workshops. In the almost two years since the Action Plan was published, seven workshops have been held (see Table 1):

- Health literacy in the education and training system;
- Importance of the media for strengthening health literacy (2 workshops);
- User-friendly and health literate health care system;
- Integrating health literacy into the care of people with chronic illness;
- Strengthening health literacy in a diverse society: Focus on migration;
- Systematic research on health literacy.

The workshops were organized and coordinated by the management office set up by the editors of the National Action Plan and followed a *scheduled pattern*:

- Short introduction to the topic, to available empirical findings and the appropriate recommendation by one of the National Action Plan editors.
- Statements by the participants on the topic of the workshop; presentation of their respective perspectives; and, from their point of view, priority aspects of implementation, followed by an initial summarizing discussion.
- Division into working groups, detailed discussion of specific sub-topics, and development of specific implementation proposals.
- Presentation of the results for implementation and detailed summary discussion with initial focus on the strategy paper.
- Summarization of the feedback round.
- Trained junior scientists documented the workshops extensively. This documentation served as the fundament for developing and preparing the strategy papers (see below), as well as data material for the evaluation of the workshop.

##### 3.3.2. Excursus: An Example Workshop

To illustrate, we examine one of the workshops here: “Integrating health literacy into the care of people with chronic illness”. The workshop was attended by stakeholders and experts from the Action Plan; from academia, politics, leading health care and social organizations; from the German Coalition for Patient Safety; and from patient and self-help organizations, as well as two experts from Austria and Switzerland.

The workshop began with a brief explanation of the most important findings on the health literacy of people with chronic illness and an introduction to the National Action Plan, especially in regard to Recommendation 11: “Integrate health literacy into the care of chronically ill” [18] (p. 41). This was followed by a presentation with short statements on Recommendation 11 by each of the participants.

It became clear that many participants had difficulty understanding the concept of health literacy. At the same time, a wide range of perspectives were brought to the fore, including critical views of the concept and its significance for people with chronic conditions. Health literacy was repeatedly presented in close connection with other concepts, such as patient education, quality management, patient safety or also inclusion. Nevertheless, specific key topics also crystalized, which led to the formation of four working groups to address them. Participants extensively discussed the recommendations in a moderated debate and deliberated concrete implementation strategies, as well as the necessary related measures. The results were then presented and collectively discussed.

Special emphasis was placed on the importance of improving the lack of information within the German health care system during chronic illness, which does not reach many patient groups. At the same time, participants also emphasized that improving information alone is not enough. What is required is to also optimize accessibility and tailor information more closely to the target groups and their everyday life. Each working group also underscored the necessity of a change in communication and cooperative interaction between health professionals and their clients, as well as their intimate others. Participants also considered more patient empowerment and enabling as important to help patients become active and co-productive. However, they also stressed that this would require a shift in power structures, which in turn would require a relearning on all sides. Difficulties in navigating the health care system and the urgency of establishing transparency and facilitating patient security were also emphasized. In addition, a strict patient-centered care as well as an orientation on illness trajectory, continuous information, and steady support were demanded.

The discussion was followed by a debate on the key points for the strategy paper that was to be drawn up. The core topics of the paper to emerge were patient-centered care, participation, relevance to everyday life, and information as a continuous task of the health care system, as well as improvement in the education, training, and development of health care professionals but also better cooperation with family members.

As usual, a strategy paper was cooperatively developed following the workshop [27], which summarized and condensed the workshop’s results into one document detailing the initial structuring and defining the hypothetical core statements. The strategy paper was drafted by the workshop leaders (editors of the Action Plan and members of the expert advisory board), commented upon by workshop participants, further developed based on revision suggestions for a period of 14 days, and finally agreed upon.

This resulted in specific strategic propositions:

- To envisage the health care system from the perspective of those living with chronic illness.
- Anchor health literacy into the everyday life of people with chronic conditions and facilitate participation.
- Offer the right information at the right time: support people with chronic conditions through a systematic health management during the entire illness trajectory.
- Understand the acquisition of information and knowledge as a learning process.
- Promote advocacy support.

The final edition of the strategy paper was published on the National Action Plan’s website and distributed. It was met with a great deal of enthusiasm, especially from patient and self-help organizations but also from organizations and associations in the health care system. Some organizations were very proud of the patient centered care emphasized in the strategy paper, as well as the program it included. This led to a considerable willingness for cooperation and implementation, which was demonstrated by a rapid increase in requests for lectures and conference participation.

The reaction to all of the strategy papers was very positive. This is demonstrated not only by the almost 3000 hits on the website (as of 25 May 2020) but also by the direct feedback received on the strategic propositions. These propositions have made a significant contribution to the debate and have frequently motivated the workshop participants to commit themselves to implementing the Action Plan.

#### 3.3.3. Cooperation with the Alliance for Health Literacy

A second important element of the collaborative implementation strategy was participation in the Alliance for Health Literacy, founded in 2017 by the Federal Ministry for Health. The most important and powerful players—the health care system’s leading associations—joined together at the initiative of the Ministry with the intention of launching and implementing projects to promote health literacy in their field of activity [28,29]. The program has made the Alliance for Health Literacy an important body for implementation of the Action Plan and its recommendations. At the same time, participation in the Alliance facilitates support of members in realizing the tasks they have been assigned, as there is a lack of vision and professional expertise. The role of the Action Plan’s experts is in consultation and the provision of specialist expertise and knowledge in the hope that in return, Alliance members will become more willing to implement the Plan and realize initiatives which will increase and strengthen health literacy.

Despite the professional support, the willingness to act remained subdued. The Alliance for Health Literacy originated from above, from the Federal Ministry of Health, with a top-down strategy which the Alliance members did not oppose, but with which they did not strongly identify and which they most certainly did not incorporate or adopt because it did not fit their current working style. There were exceptions, those who saw in the concept of health literacy the chance to strengthen the approaches already in use to improve patient information. However, the large majority remained indifferent. Furthermore, many of the associations represented in the Alliance tended to be competitors. Moreover, a third point, they did not feel sufficiently supported by their leadership and acted quasi without any real approval. Health literacy is still not a top priority today (also not politically) and has been considered a minor issue, which will eventually lead to stagnation and paralysis.

Therefore, a change in strategy was recommended: To move to a bottom-up strategy which relied on co-production, in the hope that this would awaken a willingness to act and mobilize Alliance members. To this end, the conference “Health literacy in the digital age” was to be jointly planned and hosted by the Federal Ministry of Health, the Alliance, and the Action Plan. The idea found widespread acceptance. The aim of the conference was to make the Alliance more publicly visible by having its members present relevant projects on health literacy. At the same time, the joint conference was intended to strengthen identification with the Alliance and the topic of health literacy and to convince Alliance members to advocate more strongly in support of health literacy in Germany.

The objective was met: During the complex planning phase, which took approximately ten months, Alliance members—divided into various working groups—prepared individual workshops, developed projects, discussed them, created posters and information material, and were active in a superordinate program committee. The intensive preparation contributed to an increasing identification not only with the conference but also the topic of health literacy among the participants and led to more engagement to ensure the event was a success. The fact that the leadership was included and was able to present itself in a prominent position also contributed largely to this success. With more than 300 participants, the conference garnered a high degree of attention and clearly contributed to the adoption of the topic “health literacy”. Just how sustainable the achieved effects will be remains to be seen.

Overall, the change in strategy has succeeded in anchoring the topic of health literacy in the setting of “health care system”—or better, in the Alliance for Health Literacy and its members/top organizations—and in increasing the willingness to adopt it. In the future, it will be important to stabilize this and to achieve a sustainable willingness to act.

## 4. Discussion

The National Action Plan to promote health literacy in Germany was published in 2018. The Plan has received a great deal of attention among the leading actors in the health care and education system. It already has become a reference for designing new programs in health promotion and prevention and in health communication and is well accepted among professional actors such as medical doctors, nurses, therapists, teachers, or social workers. In research, the topic of health literacy has undergone a steep thematic career; many new research projects and networks emerged here (see [30]). At the political level, the National Action Plan has succeeded in giving health literacy a prominent place on the health policy agenda; a willingness to act has been created, and a whole series of health literacy innovations have been initiated. Many of the goals of the plan have thus been achieved. The main reason for this success is the innovative format of a National Action Plan but also the implementation strategy that has been chosen.

There are, however, some limitations: It has not yet been possible to establish an alliance of all important actors in order to enable a systematic approach to execution and to create the necessary networks. Although the first networks are emerging, they exist relatively loosely side by side and require better links. This is mainly due to the fact that the formation of networks and the establishment of broad action alliances require considerable time and financial resources. This is all the more true since the implementation of extensive programs with a high innovation content, such as those linked to action plans for the promotion of health literacy, must be designed as a long-term process. Both of these aspects must be taken into account when designing and planning implementation strategies in the future.

The three-part implementation strategy, which is comprised of different scientific approaches (diffusion, dissemination, implementation), has helped to succeed. As has been demonstrated, diffusion and dissemination are absolute imperative steps to make innovative concepts and programs known and to create an interesting “atmosphere”, which stimulates curiosity and interest for the topic. Diffusion and dissemination made it possible to provide information about the National Action Plan, to communicate its relevance, and—to some extent—to exact commitment.

Overall, both steps have succeeded, as shown by the awareness of the Action Plan among stakeholders in the health sector as well as the requests for presentations and the hits of the website. Dissemination could have, however, been broader and more systematic. It also concentrated primarily upon the health care system. The education and training system, as well as other important areas of society, have been little involved. This was due to the lack of financial resources. Dissemination also came up against terminological and conceptual incompatibilities that could not have been anticipated: “Health literacy” is translated into the German “Gesundheitskompetenz” (health competence) that, surprisingly, causes a host of misunderstandings. This led to a terminological and conceptual collision between “Gesundheitskompetenz” and the German discussion on prevention and health promotion, which has again and again elicited the need for clarification.

Such incompatibilities are a frequently observed phenomenon when introducing new concepts and are also not uncommon internationally, as the concept of health literacy shows (see, e.g., [31]). As the concept became better known, terminological problems were smoothed over and resistance to its adoption diminished. At the scientific level and in research, health literacy has already become an important topic and has asserted itself as a new concept. Nevertheless, these incompatibilities should have received more attention.

*Workshops* with politicians and key stakeholders formed the core element of the implementation strategy. The workshops have proved to be successful, as the feedback rounds and the increase of requests for talks or support following each one shows. This success is demonstrated by the many health literacy projects that have been independently initiated. The identification with the strategy papers was also unexpectedly high. However, implementation of the workshops was at times laborious and time-consuming. Recruiting participants proved to be especially challenging. For example, it was very difficult to motivate and persuade politicians to take part; as such, stakeholders made up the majority of participants.

Much time was devoted at the beginning of each workshop to the terminological and conceptual difficulties mentioned above. If the workshops consisted of participants who tended to see themselves as innovators, the corresponding clarification processes were very quick, and adoption and implementation efforts for their own field of activity were rapidly considered. Other participants tended to be more hesitant. Aversion and skepticism, however, were seldom the case. During the workshops and especially during the working group sessions, it was almost always possible to diminish underlying reservations and create a positive dynamic of implementation.

At the same time, experience has shown that one-time workshops do not suffice. The workshops did produce remarkable results, as the final strategy papers show; however, singular workshops could become equivalent to an appetizer. They can promote and facilitate the willingness for innovation and possibly also encourage deliberation on implementation, but alone they are not enough to create sustainable effects. As experience confirms, a long-term, process-oriented approach is therefore indeed necessary, especially since the promotion of innovation and implementation requires time and takes place gradually [24,32]. Therefore, to achieve sustainable discussions and implementation efforts, it is necessary to continue the process that has begun and to consider holding regular workshops or specialized events. In this context, aspects of innovation management and the promotion of willingness to be innovative in the implementation process require greater attention. At the same time, a collaborative approach [21] should be strictly maintained, as it has proved to be effective.

This also applies to the cooperation with the Federal Ministry of Health’s Alliance for Health Literacy and the relevant networks—the second component of the collaborative implementation strategy. A close cooperation with the Ministry and the Alliance for Health Literacy has in fact developed, which has also survived changes in ministers and the subsequent changes in topics and has coalesced, among other forms, in a joint conference. However, whether this has led to the incorporation of health literacy as a topic and to the development of a stable alliance for the implementation of the National Action Plan is difficult to assess. The change from a top-down to a bottom-up strategy based on cooperation has undoubtably encouraged commitment; this, however, will need to be further stabilized. Thus, an ongoing process is also necessary here, one which requires further steps and further commitment. 

The following lessons learned can be summarized:

- It is necessary not only to invest into the development of action plans but also to plan the implementation followed by a systematic and scientifically sound and carefully planned dynamic implementation process.
- Diffusion and dissemination are imperative to making new innovative concepts and programs known.
- Innovations are always met with reservations. Therefore, it is important to anticipate obstacles and resistance and also to find solutions.
- Collaborative workshops with stakeholders have proven to be very important.
- Implementation requires sufficient financial and time resources as well as a specific qualification.

## 5. Conclusions

The experience to implement the National Action Plan Health Literacy in Germany demonstrates how important it is to not only develop proper strategies which promote health literacy but to also consider, systematically plan, and address the subsequent step—that of implementation. This very seldom happens in the field of health literacy. The focus is often on “natural implementation”, a strategy which, as the literature shows [32], is not particularly successful and to which numerous implementation deficits can be attributed.

With our observations, we wanted to show how important a systematic implementation and the development of scientifically founded strategies for such implementation are. At the same time, it was of special concern to us to present the three-part collaborative and co-productive approach (diffusion, dissemination, and implementation) we have chosen and to discuss it. This approach has proven feasible and should be considered for similar initiatives. In Germany, this approach has helped to place health literacy at the top of the health policy agenda.

## Figures and Tables

**Table 1:** Workshop overview.

Date	Workshop Title	Number and Type of Participants	Outcome ^1^
25 June 2018	Enabling the education and training system to promote health literacy early in life	32 participants from politics, governmental institutions, national associations, public health, foundations, academia, practical projects	Strategy paper with 4 strategic propositions for implementation
31 October 2018	Integrating health literacy into the care of people with chronic illness	29 participants from politics, leading health care and social organizations, patient and self-help organizations, academia	Strategy paper with 5 strategic propositions for implementation
12 November 2018	Facilitating the handling of health information in the media	42 participants from politics, online platforms and portals, magazines, TV productions, broadcasting agencies, governmental institutions, foundations, academia, journalism	Strategy paper with 4 strategic propositions for implementation
25 January 2019	Integrating health literacy as standard at all levels of the healthcare system	38 participants from politics, the Alliance for Health Literacy, health insurances, governmental institutions, national associations, academia, foundations	Strategy paper with 5 strategic propositions for implementation
2–3 May 2019	Systematic research on health literacy	200 national and international participants on the international symposium “Health literacy: research–practice–politics”	Strategy paper with 4 strategic propositions for implementation
20 September 2019	Strengthening health literacy in a diverse society: Focus on migration	28 participants from migrant- and self-help organizations, national associations, practical projects, academia, health care institutions	Strategy paper with 5 strategic propositions for implementation
4 February 2020	Importance of mass media for strengthening health literacy	Podium discussion with 8 journalists, 50 participants with expertise in health literacy	Conference report summarizing the results of the discussions

^1^ The strategy papers and the conference report can be downloaded at the website [26].
